# Shenqiwan Ameliorates Renal Fibrosis in Rats by Inhibiting TGF-*β*1/Smads Signaling Pathway

**DOI:** 10.1155/2017/7187038

**Published:** 2017-05-30

**Authors:** Hongshu Chen, Yiqing Xu, Yuanxiao Yang, Xiaojie Zhou, Shijie Dai, Changyu Li

**Affiliations:** ^1^The First Affiliated Hospital of Zhejiang Chinese Medical University, Hangzhou, Zhejiang 310006, China; ^2^College of Pharmacy, Zhejiang Chinese Medical University, Hangzhou, Zhejiang 310053, China; ^3^Hangzhou Medical College, Hangzhou, Zhejiang 310053, China

## Abstract

Epithelial-mesenchymal transition (EMT) refers to the transition of epithelial cells into mesenchymal cells. Emerging evidence suggests that EMT is a key point in renal interstitial fibrosis (RIF). Traditional Chinese Medicine Shenqiwan (SQW) is widely used in clinical treatment of chronic kidney disease, but the underlying mechanism remains unclear. The purpose of this study is to investigate the effect of SQW on renal fibrosis and its association with TGF-*β*1/Smads signaling pathway. A rat model of adenine (150 mg/kg) was established and intragastrically treated with various concentrations of SQW at dose of 1.5 g/kg, 3 g/kg, and 6 g/kg. Control group and model group were given the same volume of saline. Meanwhile, the positive control group was treated with Enalapril (4 mg/kg). Animals were sacrificed on 21st day after administration. The results showed that SQW could significantly relieve renal pathological damage caused by adenine, increase gene and protein expression of E-cadherin, and decrease the expression of Vimentin in kidney samples. In addition, SQW efficiently inhibited the mRNA and protein expression of p-Smad2/3 by upregulating Smad7. These results suggest that SQW could slow down the progression of renal fibrosis, possibly by inhibiting TGF-*β*1/Smads signaling pathway.

## 1. Introduction

Renal interstitial fibrosis (RIF) is the final common pathway of chronic kidney disease to the end stage renal failure. The pathological changes are characterized by inflammatory cells infiltrating, accumulation of extracellular matrix (ECM), and fibroblast proliferation [1]. Emerging evidence suggests that epithelial-mesenchymal transition (EMT) plays an important role in renal interstitial fibrosis [2, 3].

EMT is characterized by the loss of epithelial cell marker proteins including E-cadherin, cytokeratin, and an increase in expression of mesenchymal marker proteins like *α*-smooth muscle actin, Vimentin, fibroblast specific protein-1, and fibronectin [4]. Several intracellular signal transduction pathways are essential in controlling the process of EMT [5–7]. TGF-*β*1-induced EMT is particularly relevant to the pathogenesis of renal fibrosis. Smad2 and Smad3 are transcription factors that mediate the effects of TGF-*β*1 [8, 9]. Upon stimulation by TGF-*β*1, transmembrane type II TGF-*β* receptor forms tight complexes with the type I receptor, leading to phosphorylation and activation of Smad2 and Smad3. Phosphorylated Smads then heteroligomerize with the common partner Smad4 and translocate into the nucleus, where they control the transcription of TGF-*β*-responsive genes [10–12]. Hence, intervention of TGF-*β*1/Smads signaling pathway has been the most intensively targets of antifibrotic therapies.

Chinese medicine believes that “kidney-yang deficiency” is an internal condition in the development of renal fibrosis. Therefore, “tonifying the kidney-yang” is the main principle of the ancient Chinese physicians in the treatment of renal fibrosis. Shenqiwan (SQW) is one of the most classic recipes in the clinical treatment of chronic kidney disease. It was first recorded in Zhang Zhongjing's Jingui Yaolue dating back to the Eastern Han Dynasty. The prescription consists of Dihuang (Radix Rehmanniae), Danpi (Cortex Moutan), Zhuyu (Dogwood), Fulin (*Poria cocos*), Shanyao (Yam), Zhexie (Alisma), Rougui (Cassia Presl), and Fuzi (Monkshood). There are about 20 absorbed components highly correlated with the therapeutic effect of SQW against kidney-yang deficiency, especially azelaic acid-O-glucuronide, jionoside D, azelaic acid, poricoic acid B-O-sulfate, tumulosic acid-O-glucuronide, poricoic acid A, eugenol methyl ether, and dehydroeburicoic acid [13]. Previous studies have shown its antifibrotic effects in renal fibrosis [14–17]. In addition, studies also show that treatment combined with SQW is more effective than using Western medicine alone in treating chronic renal failure [18]. Our pretests also found that SQW can relieve renal pathological damage caused by adenine, but the underlying mechanism remains unclear. Therefore, the purpose of this study is to investigate the effect of SQW on renal fibrosis and its association with TGF-*β*1/Smads signaling pathway.

## 2. Materials and Methods

### 2.1. Animals and Groups

The experiments were approved by the Ethics of Committee of Zhejiang Traditional Chinese Medical University. Male SD rats in a SPF grade (age, 8 weeks; weight 200 ± 20 g) were purchased from Chinese Academy of Sciences of Shanghai Laboratory Animal Center and housed at a constant room temperature (20 ± 2°C) and supplied with sterilized food and water. The rats were subjected to acclimatization for a week before the experiment. Totally 60 SD rats were randomly divided into the control group (*n* = 10), model group (*n* = 10), SQW group (1.5 g/kg/d, 3 g/kg/d, and 6 g/kg/d; *n* = 10 in each dose group), and Enalapril group (4 mg/kg/d; *n* = 10). Except control group, the groups were all treated with adenine (150 mg/kg/d) for three weeks. Then, the rats of drug groups were intragastrically administration with SQW at a dose of 1.5 g/kg/d, 3 g/kg/d, and 6 g/kg/d or Enalapril at a dose of 4 mg/kg/d. The control group and model group were intragastrically with the same volume of saline. The rats in each group were sacrificed on day 21 after treating. Biochemical indicators samples and organs were harvested for analysis. The rest part of them were stored at −80°C for RNA and protein extraction.

### 2.2. Biochemical Measurements in Blood and Urine

The blood samples, acquired from the heart after 21 days treatment, were centrifuged at 3000 rpm for 10 min (4°C) and the collected 24 h urine samples were centrifuged at 3000 rpm for 5 min (4°C). All of the samples were stored at −80°C. Serum creatinine (Scr), blood urea nitrogen (BUN), and 24 h urinary protein levels were measured by automatic biochemical detector (Hitachi Co., Tokyo, Japan).

### 2.3. Renal Pathological Studies

The kidney tissues were fixed in 4% paraformaldehyde. After embedded in paraffin, the renal tissues were cut into slices, sections were stained with HE and PAS to evaluate renal structural injury, and Masson staining was stained to observe the degree of renal fibrosis. Sections were randomly chosen under the microscope (Leica, Wetzlar, Germany) on high-power fields and photographed in each group.

### 2.4. Immunocytochemistry Studies

Paraffin-embedded renal sections (3–5 *μ*m) were taken for immunohistochemical analysis. The sections were quenched with 3% H_2_O_2_ in 10 min, washed by PBS for 3 times, and then incubated overnight with anti-E-cadherin primary antibodies (ab76055, Abcam, Cambridge, UK) and anti-Vimentin primary antibodies (ab45939, Abcam, Cambridge, UK). Subsequently, the stained sections were incubated with peroxidase conjugated secondary antibodies. The immunocomplexes were visualized by DAB substrate. The sections were all counterstained with hematoxylin before mounting. We judged the positive expression intensity according to the staining area in each field and image analysis was performed by Image Pro Plus software.

### 2.5. Reverse Transcription-Quantitative Polymerase Chain Reaction (RT-qPCR)

Total RNA from the renal tissue samples were extracted using the AxyPrep RNA Kits according to the manufacturer's manual. Then PrimeScript RT reagent kits (TaKaRa Biotechnology) were used to synthesise cDNA. The specific primers for the renal tissue samples are listed in Table 1. The conditions of PCR cycling were as follows: predenaturation at 95°C for 3 min, 48 cycles at 95°C for 10 sec, and 60°C for 30 sec. The quantity of mRNA was calculated through the cycle threshold (CT) values which was calculated by the computer software. The relative mRNA expression levels were determined by 2^−ΔΔCt^ method.

### 2.6. Western Blot Analysis

Total proteins from nephridium were extracted with RIPA lysis buffer and then equal amount of these proteins was resolved in SDS-PAGE gel and transferred onto PVDF membrane; the membranes were incubated overnight with antibodies after blocking with 5% nonfat milk. The antibodies were used as follows: TGF*β*1 (ab64715, Abcam, Cambridge, UK), anti-E-cadherin (ab76055, Abcam, Cambridge, UK), anti-Vimentin (ab45939, Abcam, Cambridge, UK), anti-Smad2/3 (ab63672, Abcam, Cambridge, UK), anti-p-Smad2/3 (ab63399, Abcam, Cambridge, UK), anti-Smad7 (ab90086, Abcam, Cambridge, UK), and *β*-actin (Santa Cruz Biotechnology, Inc.). After that the membranes were incubated with peroxidase conjugated secondary antibodies. The optical density of bands was quantified with *β*-actin as an internal inference and the immunocomplex was visualized with Odyssey near infrared dual color laser imaging system.

### 2.7. Statistical Analysis

Data were showed as means ± standard deviation (SD). We adopted GraphPad Prism 5.0 software and SPSS software (Version 17.0) for statistical analysis. Student's *t*-test was taken to compare between the two groups and ANOVA was used in multiple groups to assess statistical significance. *P* < 0.05 or *P* < 0.01 was considered significantly difference.

## 3. Results

### 3.1. SQW Decreases the Level of BUN, Scr, and U-Pro/24 h in Rats

**Figure 1:**
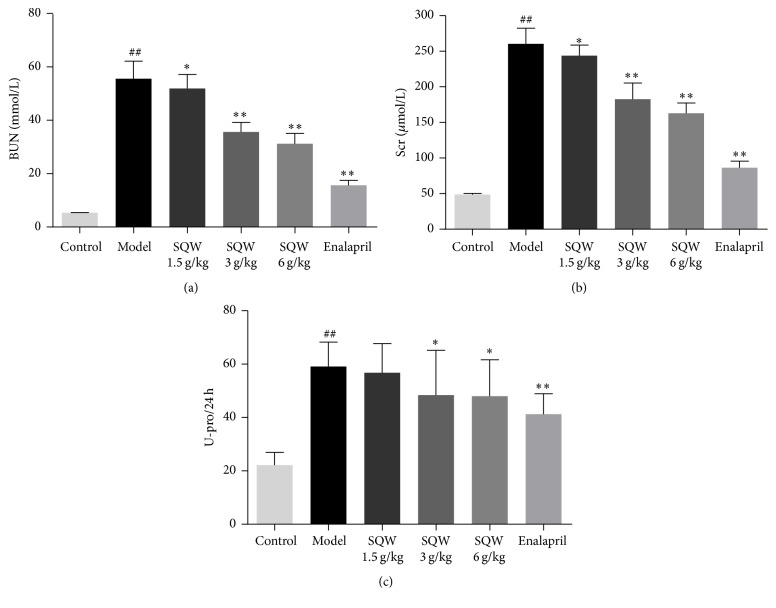
The Effect of SQW on the levels of BUN, Scr, and U-pro/24 h in Rats. ^##^*P* < 0.01 compared with control group; ^*∗*^*P* < 0.05; ^*∗∗*^*P* < 0.01 compared with model group.

As shown in Figure 1, BUN, Scr, and U-pro/24 h were significantly elevated in model group compared with the control group (*P* < 0.01). After 21 days of given SQW, the indicators above were all reduced. The expression of BUN and Scr was decreased in dose-dependent (*P* < 0.01). The level of U-pro/24 h was obviously reduced in middle and high-dose group of SQW except low-dose group (*P* < 0.05). The result indicated that kidney injury model had been successfully established by oral administration of adenine and SQW could protect renal injury.

### 3.2. SQW Alleviates Kidney Tissue Injury in Rats

**Figure 2:**
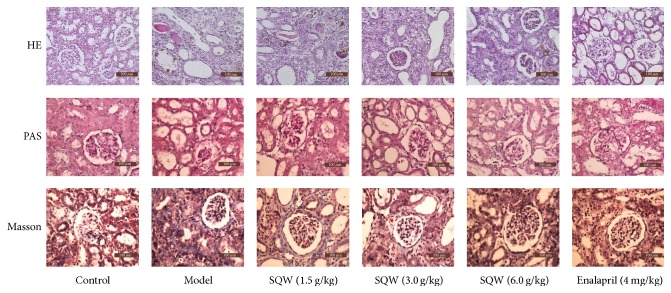
Effect of SQW on renal histopathology in rats induced by adenine. HE and PAS staining were all showed the renal structural damage in rats after oral administration of adenine (200x magnification). Masson staining illustrated the degree of renal fibrosis in rats (200x magnification).

HE and PAS staining showed the morphological changes in the model group including renal tubules ectasia, inflammatory cell infiltration, glomerular necrosis, and segmental thickening of glomerular basement membranes (Figure 2). However, after given SQW, these abnormalities were all alleviated. As shown in Masson staining, we could observe that fibrosis area and collagen deposition were decreased in SQW groups, indicating that SQW could lighten kidney renal fibrosis.

### 3.3. SQW Ameliorates Renal Fibrosis though Increasing the Expression of E-Cadherin and Decreasing Vimentin Expression in Rats

**Figure 3:**
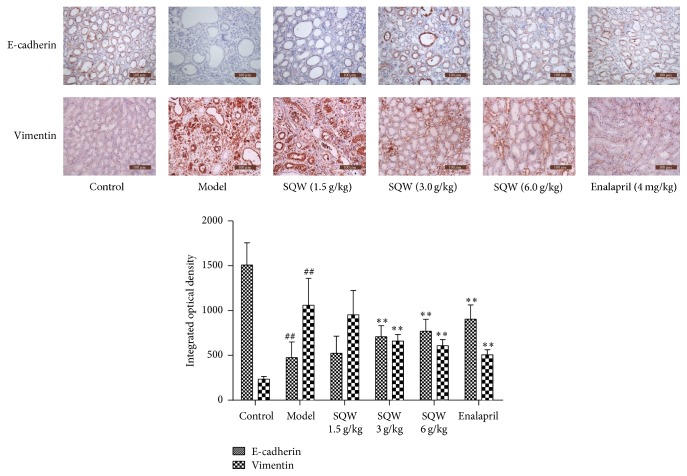
Effect of SQW on the expression of E-cadherin and Vimentin in kidney samples (200x magnification). ^##^*P* < 0.01 compared with control group; ^*∗∗*^*P* < 0.01 compared with model group.

The result of immunohistochemistry (Figure 3) demonstrated that the expression of Vimentin was increased and E-cadherin expression was decreased in the model group (*P* < 0.01), and indicators above were both partly reversed after treating with SQW. The changes were obvious in the middle and high-dose group of SQW (*P* < 0.01), proving that SQW could alleviate kidney tissue fibrosis in rats.

### 3.4. SQW Ameliorates Renal Interstitial Fibrosis by Suppressing the TGF-*β*1/Smads Signaling Pathway in Kidney Tissue

**Figure 4:**
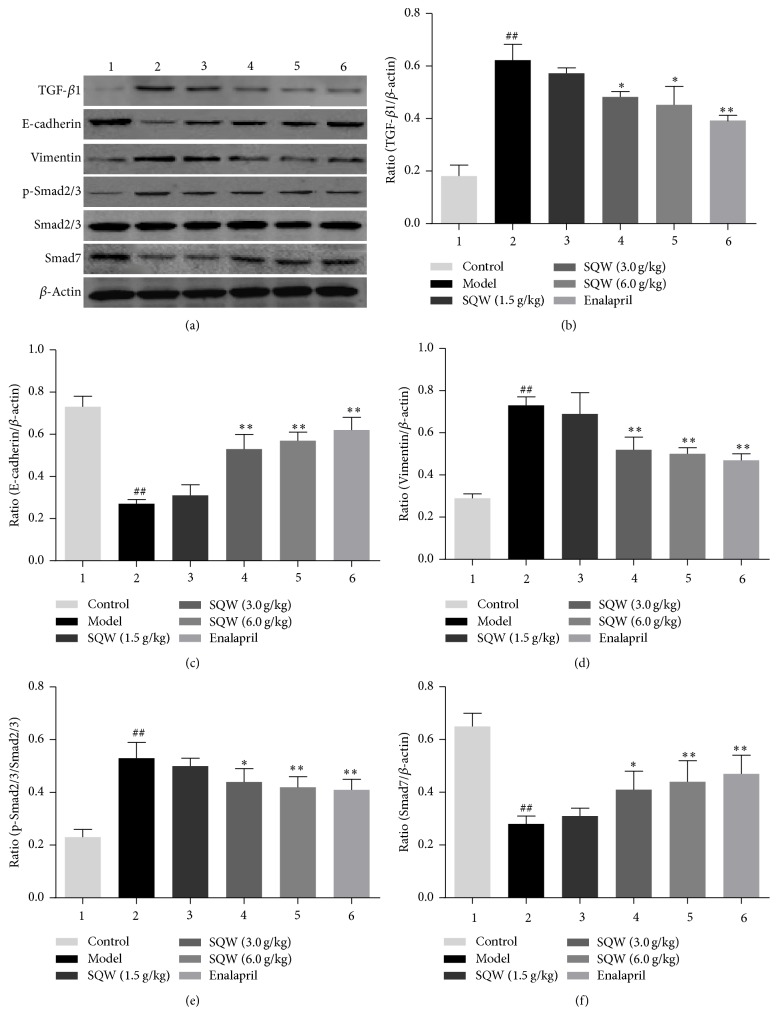
Effect of SQW on expressions of TGF-*β*1, E-cadherin, Vimentin, p-Smad2/3, and Smad7 detected by Western blot in kidney samples. Semiquantitative data of the proteins were presented as relative ratio to *β*-actin. Semiquantitative data of p-Smad2/3 were presented as the relative ratio to Smad2/3. ^##^*P* < 0.01 compared with control group; ^*∗*^*P* < 0.05; ^*∗∗*^*P* < 0.01 compared with model group.

**Figure 5:**
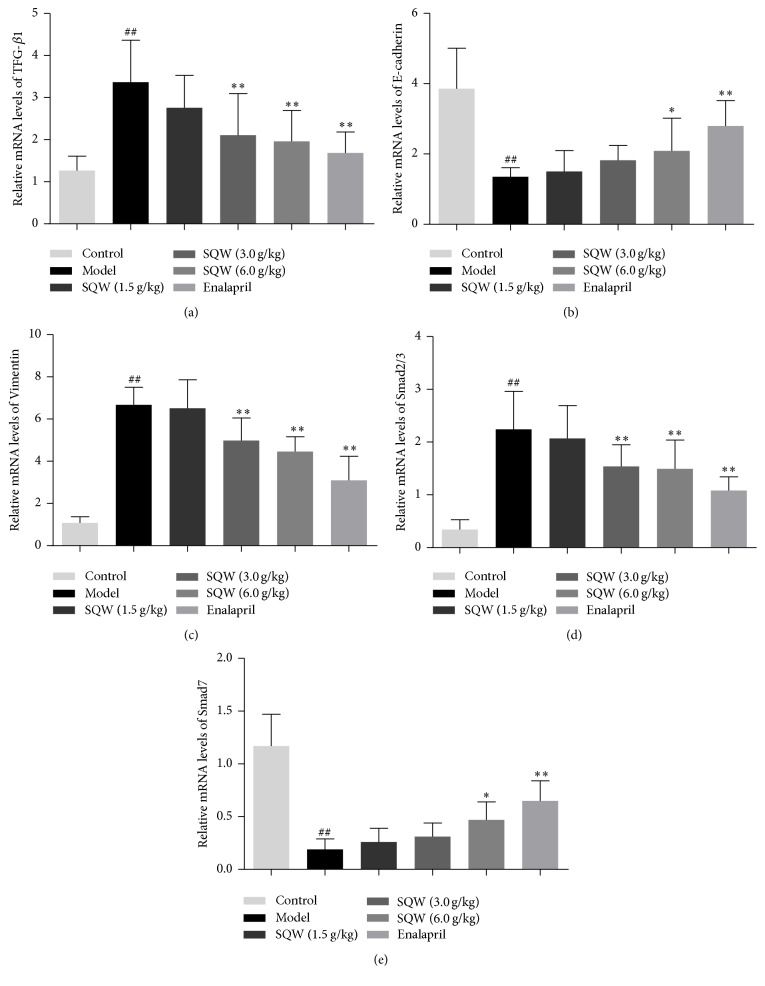
Effect of SQW on relative gene expressions of TGF-*β*1, E-cadherin, Vimentin, Smad2/3, and Smad7 mRNAs detected by RT-PCR in kidney samples. ^##^*P* < 0.01 compared with control group; ^*∗*^*P* < 0.05; ^*∗∗*^*P* < 0.01 compared with model group.

Compared with the model group, the expression of TGF-*β*1 and Vimentin was significantly downregulated in SQW group (Figures 4(a), 4(b), and 4(d)), which were similar to the mRNA expression of them (Figures 5(a) and 5(c)). In order to further demonstrate the role of SQW in the process of EMT, we measured the genes and proteins expression of Smad2/3, p-Smad2/3, and Smad7 in TGF-*β*1/Smads signaling pathway. As shown in Figures 4(a), 4(e), 4(f), 5(d), and 5(e), the ratio of p-Smad2/3 to Smad2/3 was decreased in the SQW-treated groups. The expression of Smad7 was upregulated in middle and high-dose group. In addition, the mRNA expression of Smad2/3 and Smad7 genes in SQW group was similar to the presentation of proteins above. From this we could know that SQW could improve renal fibrosis probably through inhibiting TGF-*β*1/Smads signaling pathway.

## 4. Discussion

The latest national survey shows that chronic kidney disease (CKD) has become a serious health problem in China [9, 19–21]. And no matter in animal studies or in clinical patients, chronic kidney disease presents the primary pathological feature of renal interstitial fibrosis. Therefore, the prevention of renal interstitial fibrosis will be meaningful to slow down the progression of chronic kidney diseases. Studies show that traditional Chinese medicine and compounds extracted from them possess the potential effect of antifibrotic properties [22–26]. In this study, we investigate the effect of SQW on renal fibrosis and its association with TGF-*β*1/Smads signaling pathway.

HE and PAS staining showed the morphological changes in the model group including renal tubules ectasia, inflammatory cell infiltration, glomerular necrosis, and segmental thickening of glomerular basement membranes. In addition, as shown in Masson staining, we could observe that fibrosis area and collagen deposition were presented in model group. Pathological changes in the kidney of rats showed that the experiment model would be successful. These morphological changes were alleviated after being treated with SQW. The results indicated that SQW could relieve renal pathological changes in rats.

Multiple mechanisms are involved in the processing of renal interstitial fibrosis. EMT, recognized as a typical event, have gained high attention among these mechanisms [27–29]. EMT is a process that epithelial cells differentiate into myofibroblasts. This conversion is characterized by activation of fibrotic features including expressing *α*-smooth muscle actin, Vimentin, fibroblast specific protein-1, and accumulation of extracellular matrix [30, 31], inhibiting the expression of epithelial genetic phenotype such as E-cadherin. In this experiment, we observed that the gene and protein expression of E-cadherin in the model group were distinctly decreased and Vimentin was dramatically increased. In addition, partial changes can be reversed after treated with SQW.

TGF-*β*1/Smads signaling pathway is considered to be the most classic signaling pathway in renal interstitial fibrosis [32–36]. Its transduction is regulated by a mechanism that depends on Smads to transfer the stimulus from extracellular into the nucleus. The current evidence shows that inhibiting TGF-*β*1/Smads signaling by downregulating Smad2/3 and upregulating Smad7 may be an effective therapy of renal interstitial fibrosis [37–39]. Smad7 which has the ability of regulating TGF-*β*1/Smads signaling pathway serves as an autoregulatory negative feedback signal. Smad7 inhibits the phosphorylation of Smads, so that it prevents the transcription of target genes through competitive binding with TGF-*β*1/Smads. In this experiment, the expression of p-Smad2/3 was significantly decreased in SQW group. In contrast, the gene and protein expression of Smad7 were significantly increased after treated with SQW.

Therefore, we could know that SQW played a renal protective role in renal fibrosis possibly by inhibiting TGF-*β*/Smads signaling pathway. Other potential molecular mechanisms should be explored in further investigation for clarifying the exact mechanism of SQW in alleviating renal fibrosis. Undoubtedly, it is a meaningful and challenging event.

## 5. Conclusion

The present study revealed that SQW could slow down the progression of renal interstitial fibrosis, possibly by inhibiting TGF-*β*/Smads signaling pathway.

## Figures and Tables

**Table 1:** List of primer sequences for RT-qPCR.

Gene	Forward primer	Reverse primer
TGF-*β*1	5′-GACCTGGGTTGGAAGTGGAT-3′	5′-CGGGTTGTGTTGGTTGTAGA-3′
E-cadherin	5′-TCCCACTCTACCTACCAGTCTG-3′	5′-CAGTTCATCACATCACAGCACT-3′
Vimentin	5′-CTGGAGTCACTTCCTCTGGTT-3′	5′-CACCTGTCCGTCTCTGGTTT-3′
Smad2/3	5′-TCCACCAGGCTGTAATCTGAAGA-3′	5′-GACATGCTTGAGCAACTGACT-3′
Smad7	5′-GCTGGTACAGAAAGTGAGGAG-3′	5′-GTCCGGGTTGTCCAGTGTG-3′
*β*-Actin	5′-GCTCTCTTCCAGCCTTCCTT-3′	5′-GGTCTTTACGGATGTCAACG-3′
